# Accessory and Expiratory Muscles Activation During Spontaneous Breathing Trial: A Physiological Study by Surface Electromyography

**DOI:** 10.3389/fmed.2022.814219

**Published:** 2022-03-10

**Authors:** Matteo Pozzi, Emanuele Rezoagli, Alfio Bronco, Francesca Rabboni, Giacomo Grasselli, Giuseppe Foti, Giacomo Bellani

**Affiliations:** ^1^Department of Medicine and Surgery, University of Milano-Bicocca, Monza, Italy; ^2^Department of Emergency and Intensive Care, San Gerardo Hospital, Monza, Italy; ^3^Department of Anesthesia, Intensive Care and Emergency, Fondazione IRCCS Ca' Granda Ospedale Maggiore Policlinico, Milan, Italy; ^4^Department of Pathophysiology and Transplantation, University of Milan, Milan, Italy

**Keywords:** electrical activity, non-invasive surface electromyography, weaning, inspiratory muscles, expiratory muscles, spontaneous breathing trial (SBT)

## Abstract

**Background:**

The physiological and prognostical significance of accessory and expiratory muscles activation is unknown during a spontaneous breathing trial (SBT). We hypothesized that, in patients experiencing weaning failure, accessory and expiratory muscles are activated to cope with an increased respiratory workload.

**Purpose:**

To describe accessory and expiratory muscle activation non-invasively by surface electromyography (sEMG) during an SBT and to assess differences in electrical activity (EA) of the inspiratory and expiratory muscles in successful vs. failing weaning patients.

**Methods:**

Intubated patients on mechanical ventilation for more than 48 h undergoing an SBT were enrolled in a medical and surgical third-level ICU of the University Teaching Hospital. Baseline characteristics and physiological variables were recorded in a crossover physiologic prospective clinical study.

**Results:**

Of 37 critically ill mechanically ventilated patients, 29 (78%) patients successfully passed the SBT. Rapid shallow breathing index (RSBI) was higher in patients who failed SBT compared with the successfully weaned patients at baseline and over time (group-by-time interaction *p* < 0.001). EA of both the diaphragm (EAdi_surf_) and of accessory muscles (ACC_surf_) was higher in failure patients compared with success (group-by-time interaction *p* = 0.0174 and *p* < 0.001, respectively). EA of expiratory muscles (ESP_surf_) during SBT increased more in failure than in weaned patients (group-by-time interaction *p* < 0.0001).

**Conclusion:**

Non-invasive respiratory muscle monitoring by sEMG was feasible during SBT. Respiratory muscles EA increased during SBT, regardless of SBT outcome, and patients who failed the SBT had a higher increase of all the inspiratory muscles EA compared with the patients who passed the SBT. Recruitment of expiratory muscles—as quantified by sEMG—is associated with SBT failure.

## Introduction

Difficult weaning from the mechanical ventilation leads to the prolonged intensive care unit (ICU) stay and increased mortality (1). Respiratory muscles dysfunction is a common determinant of difficult weaning, and the reduction of the force-generating capacity of the diaphragm can be observed already after a few days of mechanical ventilation (2). So far, different techniques are available to assess diaphragm strength and its potential dysfunction (3).

However, the respiratory effort is variably distributed among different respiratory muscles in addition to the diaphragm, according to the inspiratory load and muscle fatigue (4). Despite this, the role of extra diaphragmatic muscles, such as accessory and expiratory muscles, has been seldom considered and this might be a relevant limitation to understand the mechanism behind weaning failure.

First, expiratory muscles recruitment has been demonstrated recently by Doorduin et al. (5), who observed that expiratory muscles recruitment progressively increases during a spontaneous breathing trial (SBT) in weaning failure patients, while remaining stable in successfully extubated patients.

Second, accessory muscles recruitment is a well-known clinical landmark of respiratory fatigue. The progressive activation of accessory muscles has been variably observed in response to an increased respiratory load (6–8). Therefore, it can be postulated that accessory muscles are recruited to cope with an imbalance between the work of breathing and the force-generating capacity of the diaphragm, although the interplay among different inspiratory muscles and their role on outcome are not yet elucidated.

Furthermore, the activation of other force-generating muscles might alter the calculation of muscular pressure (Pmus) based on electrical activity of the diaphragm (EAdi) (9) and indexes of neuromechanical coupling, such as neuroventilatory efficiency (NVE) (10).

Surface electromyography (sEMG) is a non-invasive system that allows to monitor the EA of respiratory muscles other than the diaphragm.

Surface electromyography has been applied in previously physiological studies to explore the relationship between accessory muscles Electrical Activity (EA) and respiratory load (6, 7, 11, 12) subjective dyspnea (8), and patient-ventilator asynchrony (13). Although this technique bears some technical constrains (14), we recently demonstrated that sEMG can be reliably used to measure surface EAdi that well correlates with inspiratory effort measured by esophageal pressure (15).

The success of a SBT is considered a reliable indicator of ventilator independence. Furthermore, beyond classical failure criteria (16) the physiological and prognostic significance of accessory and expiratory muscles activation during SBT is not known. Accordingly, we hypothesized that accessory muscles are activated during the weaning process in response to the increased respiratory load in weaning failure patients.

The aim of this work was to describe the accessory muscles activation by sEMG during an SBT and to assess differences in EA of the inspiratory muscles in successful vs. failing weaning patients. In addition, we explored expiratory muscles activation during expiration after stratification by success vs. failure of SBT.

## Patients and Methods

### Study Design and Population

This longitudinal study was conducted in a general 8-bed ICU of a University hospital (San Gerardo Hospital, Monza, Italy). Intubated patients on mechanical ventilation for more than 48 h were screened and considered eligible for the study if they were judged ready for an SBT, according to the prespecified safety criteria (17).

Exclusion criteria were (a) age < 18 years, (b) presence of a neuromuscular disease, (c) suspected or confirmed phrenic nerve lesion, and (d) ongoing extracorporeal membrane oxygenation (ECMO).

Informed consent was obtained according to the recommendations of our Institutional Review Board (San Gerardo Hospital Ethical Committee), which approved the study protocol.

### Study Protocol

All the patients, at the time of the enrollment, were on pressure support (PS) ventilation, set according to the attending physician decision.

The study protocol was divided into the following two phases:

1. Baseline step: Lasting 10 min during which patients were studied with the baseline ventilator setting of PS ventilation.
2. SBT: Patients were switched to Continuous Positive Airway Pressure (CPAP) at unchanged levels of Positive End-Expiratory Pressure (PEEP) and Fraction of Inspired Oxygen (FiO_2_) (standard SBT in our center) for 2 h unless failure criteria were met.

Criteria for SBT failure were:

(a) Respiratory Rate (RR) > 35 breaths/min for more than 3 min; (b) a peripheral oxygen saturation (SpO_2_) < 90%, (c) a Heart Rate (HR) > 140 beats/min or a change > 30% of the baseline value; (d) a Systolic Blood Pressure (SBP) < 90 mmHg or SBP > 180 mmHg or a change > 30% of baseline SBP; (e) an increase in End-Tidal CO_2_ (EtCO_2_) > 8 mmHg above the baseline EtCO_2_; and (f) the presence of anxiety, diaphoresis or agitation.

### Signal Recording

**Figure 1:**
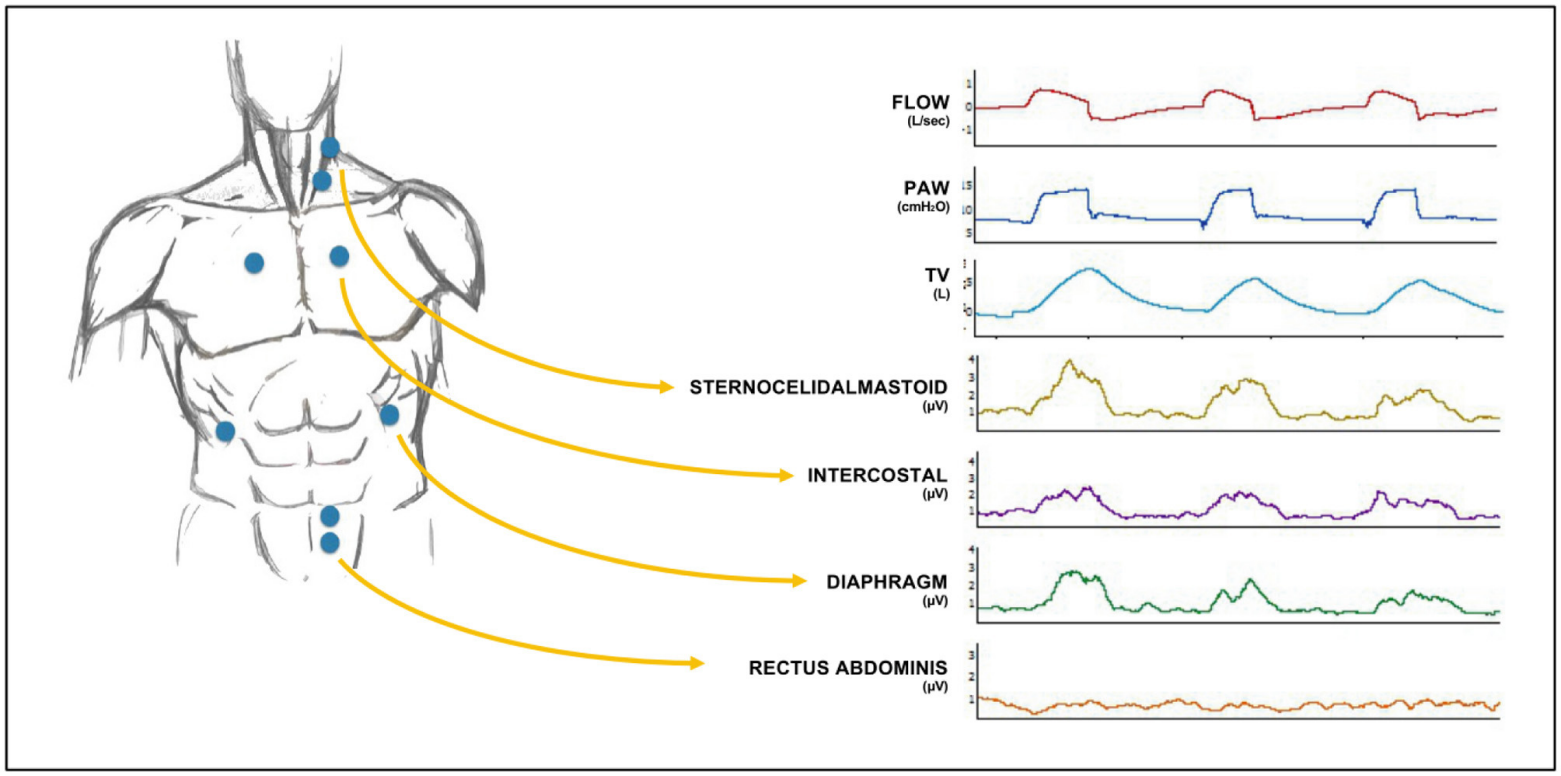
Electrode positioning on the chest wall **(left)** and general setup of the channel recordings **(right)** along with sample ventilatory waveforms and surface electromyography (sEMG) traces. The four pairs of electrodes were positioned (1) on the lower coastal margin, bilaterally on the midclavicular line, for diaphragm electrical activity (EAdi_surf_), (2) on the second intercostal space, bilaterally on the parasternal line, for parasternal external intercostal muscles EA, (3) on the middle third of the sternocleidomastoid muscle, for sternocleidomastoid EA and (4) on the left midclavicular line, at the level of the umbilicus, for rectus abdominis muscles EA. PAW, Airway Pressure; TV, Tidal Volume.

Surface electromyography from respiratory muscles and ventilator waveforms were continuously recorded during the study. The sEMG signals were collected through 4 pairs of surface electrodes (Kendall 530 Foam Electrodes, Covidien, Dublin, Ireland) positioned as follows (Figure 1):

1. Lower costal margin, bilaterally on the midclavicular line, for the costal diaphragm Electrical Activity (EAdi_surf_).
2. Second intercostal space, bilaterally on the parasternal line, for intercostal muscles and middle third of the sternocleidomastoid muscle, on the right or left side (usually opposite to the central venous line). These two signals were summed to calculate the “sum of accessory muscles electrical activity (ACC_surf_).”
3. On the left midclavicular line, at the level of the umbilicus, for rectus abdominis muscles (EXP_surf_).
4. Signals from the whole inspiratory muscles (Accessory Muscles and Diaphragm) were summed to calculate the “sum of inspiratory muscles electrical activity” (INSP_surf_). For this, on a breath-to-breath basis, INSP_surf_ represents the sum of ACC_surf_ and EAdi_surf_ absolute values.

The sEMG electrodes were connected to the Dipha 16 device (Inbiolab, Groningen, Netherlands), which amplified, prefiltered between 0 and 200 Hz, sampled at 500 Hz, and then wirelessly transmitted the EMG signals to a dedicated acquisition system, which also collected outputs from the ventilator (i.e., airway pressure and flow). Additional details about signal processing are provided elsewhere (15). Briefly, the raw surface EMG signal was filtered and cleaned of ECG artifacts by a gating procedure. The envelope of the root mean square of the signal over a 250 ms period was taken as surface EMG signal. Data were continuously recorded during the study and were stored on a hard drive for offline analysis by dedicated software (Dipha Software, Draeger Medical, Luebeck, Germany, UK).

### Data Analysis

Traces were analyzed offline by using LabChart (ADInstruments, Sydney, Australia). For every patient, 40 respiratory cycles during baseline and clusters of 20 respiratory cycles in 5 min-periods at 0, 15, 30, 45, 60, 75, 90, 105, and 120 min were manually selected. The mean and maximum values of the 4 sEMG channels were calculated during both the inspiration and expiration, that were automatically defined according to the airway flow direction. Tidal volume (TV) and maximum airflow were derived from the ventilator waveforms.

Indeed, for each respiratory cycle:

1. Diaphragm NVE (NVE_EAdi_) was calculated as the ratio between TV and the peak of EAdi_surf_.
2. Analogously, to take into account the contribution of accessory muscles, NVE_sum_ was calculated as the ratio between TV and INSP_surf_.
3. The “accessory/diaphragm electrical activity ratio” (A/D_ratio_) was calculated as the ratio between EAdi_surf_ and ACC_surf_, both considered as values normalized to baseline (see below) to quantify accessory muscles recruitment.

Further details on data analysis are provided in the Supplementary Material.

Finally, in order to counterbalance the intersubject variability of each signal EAdi_surf_, ACC_surf_, INSP_surf_, EXP_surf_, RR, TV, and Rapid Shallow Breathing Index were normalized to the baseline value of each patient and then subsequently expressed as the ratio of baseline.

### Statistical Analysis

Continuous variables were reported as median with 25th to 75th interquartile range (IQR). Categorical data were reported as count (proportion). A *p* < 0.05 (two-tailed) was considered as statistically significant. Patients were stratified based on the success vs. failure of SBT. Differences among continuous variables were tested by an unpaired Student's *t*-test or non-parametric Wilcoxon test for normally distributed variables. The change of the sEMG signal over 2 h of SBT (time) by success vs. failure of weaning (group) was tested by a mixed-effects model in which SBT outcome, time, and the interaction among two were defined as fixed effects while patients were considered as random effect. A *post-hoc* multiple comparison between the 2 groups at each time point was performed using the Tukey test. Statistical analysis was performed by SPSS software version 21 (SPSS Incorporation, Chicago, Illinois, USA).

This study was conducted and reported based on the Strengthening the Reporting of Observational Studies in Epidemiology (STROBE) guidelines for observation.

## Results

A total of 38 patients were enrolled in this study. After enrollment, one patient was excluded because of a poor sEMG signal. Among 37 patients included into the final analysis, 29 (78%) patients passed the SBT and were successfully weaned. A total of 7,297 respiratory cycles were sampled and included into the analysis. A detailed depiction of sampled respiratory cycles for each patient is provided in the Supplementary Material.

Before the initiation of the SBT, patients had been on mechanical ventilation for 10 ± 15 days with a mean PEEP of 7.7 ± 1.1 cm H_2_O and a mean PS level of 6.5 ± 2.2 cm H_2_O. After stratification according to SBT outcome, male sex was more represented among successfully weaned patients. In the same way, a trend toward a higher prevalence of chronic obstructive pulmonary disease (COPD) was observed (Table 1).

**Table 1:** Baseline characteristics of the study population were stratified by success vs. failure of a spontaneous breathing trial (SBT). Data are referred to the study day.

	**Whole population (*n =* 37)**	**SBT success (*n =* 29)**	**SBT Failure (*n =* 8)**	**P**
**Demographic characteristics**				
Sex, males, *n* (%)	27 (73%)	23 (79%)	4 (50%)	<0.001
Age, yrs	56 (50–62)	60 (51–75)	52 (41–65)	0.083
BMI, kg/m^2^	24 (23–27)	24 (23–27)	24 (21–26)	0.436
**Comorbidities**				
COPD, *n* (%) Diabetes, *n* (%) Atrial fibrillation, n (%) CAD, *n* (%) Malignancy, *n* (%) AIDS, *n* (%) Psychiatric disease, *n* (%)	5(13.5%) 2 (5.4%) 5 (13.5%) 1 (2.7%) 2 (5.4%) 2 (5.4%) 1 (2.7%)	2 (6.4%) 1 (3.4%) 3 (10.3%) 1 (3.4%) 1 (3.4%) 1 (3.4%) 0 (0%)	3 (37.5%) 1 (12.5%) 2 (25%) 0 (0%) 1 (12.5%) 1 (12.5%) 1 (12.5%)	0.0670 0.2831 0.5944 0.5944 0.3614 0.3162 0.0536
**Admission diagnosis**				
ARDS, *n* (%) Septic shock, *n* (%) Postoperative, *n* (%)	29 (78%) 6 (16%) 2 (5.4%)	23 (79%) 5 (17.2%) 1 (3.4%)	6 (75%) 1 (12.5%) 1 (12.5%)	0.7932 0.0034 0.0056
**Clinical characteristics at SBT start**				
SAPSII	47 (38–62)	47 (39–62)	47 (37–58)	1.0
SOFA	8 (5–11)	8 (5–9)	10 (4–14)	0.497
MV, days	2 (1–12)	1 (1–12)	7 (2–13)	0.524
Sedation, days	4 (2–8)	6 (2–21)	9 (5–17)	0.564
NMBA, days	1 (0–7)	1 (0–6)	7 (2–11)	0.220
AMV, days	4 (2–8)	4 (2–10)	4 (2–6)	0.470
Cpl, rs, ml/cmH_2_O	59 (40 - 65)	54 (40–65)	60 (35–68)	0.689
NIF, cmH_2_O	35 (25–36)	35 (25–41)	30 (19–34)	0.237
PaO_2_/FiO_2_, mmHg	270 (195–314)	270 (195–214)	254 (198–329)	0.862
pH	7.44 (7.41–7.45)	7.44 (7.41–7.45)	7.43 (7.42–7.45)	0.743
PEEP, cmH_2_O	8 (7–8)	8 (6–8)	8 (8–9)	0.086
PS level, cmH_2_O	6 (5–8)	6 (4–8)	7 (6–9)	0.137
RR, breaths/min	20 (16–25)	20 (16–23)	22 (18–26)	<0.0001
TV, mL	400 (370–550)	457 (397–595)	437 (383–539)	0.002
RSBI, breaths/min/L	44 (32–58)	42 (29–58)	52 (36–68)	<0.0001

*BMI, Body Mass Index; COPD, Chronic Obstructive Pulmonary Disease; AIDS, Aquired Immunodeficiency Syndrome; ARDS, Acute Respiratory Distress Syndrome; CAD, Coronary Arthery Disease; SAPS, Simplified Acute Physiology Score; SOFA, Sequential Organ Failure Assessment; MV, Mechanical Ventilation; NMBA, Neuromuscular Blocking Agent; AMV, Assisted Mechanical Ventilation; Cpl,rs, Compliance of the Respiratory System; NIF, Negative Inspiratory Force; paCO2, Arterial Partial Pressure of Carbon Dioxide*.

### Respiratory Pattern and Blood Gas Analysis During SBT

At baseline, patients who failed the SBT had a lower TV (457; 397–595 vs. 437; 383–539 ml, *p* = 0.002), a higher RR (22; 18–2 vs. 20; 16–23 breaths/min, *p* < 0.001) compared with patients who were successfully weaned. This led to a higher baseline rapid shallow breathing index (RSBI) in patients who failed SBT compared with patients who passed it (42; 29–58 vs. 52; 36–68 breaths/min/L; *p* < 0.0001) (Table 2). RSBI increased during SBT (*p* < 0.0001 for the effect of time) and the rise was higher in the failure group (*p* < 0.0001 for the interaction of time and SBT outcome). P0.1 remained stable through SBT with no influence on SBT outcome (Table 3). EtCO_2_ increased over time during the trial (*p* = 0.003) regardless of the SBT outcome (Table 3).

**Table 2:** Respiratory mechanics variables and neuroventilatory efficiency of diaphragm and accessory inspiratory muscles by weaning success vs. failure during of the SBT.

	**Whole population (*n =* 37)**	**SBT success (*n =* 29)**	**SBT failure (*n =* 8)**	***p***
RR, breaths/mL	21.3 (17.9–26)	20.8 (16.5–25)	23.9 (20.8–27.9)	<0.001
TV, mL^*^	415 (363–544)	437 (363–569)	397 (361–499)	0.1166
RSBI, L/breaths/min^*^	52.2 (36.6–68.6)	48.3 (30.9–67.7)	57.2 (46.2–77.4)	0.0025
NVE_EAdi_ (L/μV)^*^	0.121 (0.085–0.184)	0.127 (0.086–0.198)	0.110 (0.083–0.143)	<0.0001
NVE_sum_ (L/μV)^*^	0.047 (0.029–0.66)	0.049 (0.030–0.071)	0.042 (0.026–0.052	<0.0001
NVEacc (L/μV)^*^	0.082 (0.045–0.130)	0.080 (0.048–0.144	0.072 (0.039–0.092	<0.0001
A/D${}_{\text{ratio}}^{*}$	0.83 (0.56–1.10)	0.825 (0.553–1.117	0.867 (0.590–1.093	0.0697

*RR, respiratory rate; TV, tidal volume; RSBI, rapid shallow breathing index; NVE_EAdi_, neuroventilatory efficiency of the diaphragm; NVE_sum_, neuroventilatory efficiency of the accessory muscles. A/D ratio*;

* *All measurement was average through the entire SBT period (i.e. 2 h)*.

**Table 3:** Respiratory variables at three time points in the whole population.

	**Baseline**	**1^**st**^ h**	**2^**nd**^ h**	***p***
P0.1, cmH_2_O	1.2 (0.9–1.8)	1.6 (1.4–2.5)	1.9 (1.05–2.65)	*p* = 0.6525 (time) *p =* 0.6214 (SBT outcome) *p =* 0.8939 (interaction)
Compliance, mL/cmH2O	59 (40–65)	65 (40–70)	58 (40–71)	*p =* 0,0002 (time) *p =* 0.4720 (SBT outcome) *p =* 0.6698 (interaction)
P/F, mmHg	270 (195–314)		264 (227–289)	*p =* 0,8226 (time) *p =* 0.4457 (SBT outcome) *p =* 0.8530 (interaction)
pCO_2_, mmHg	44 (41- 48)		45 (40–47)	*p =* 0,6685 (time) *p =* 0.8021 (SBT outcome) *p =* 0.4514 (interaction)
pH	7,44 (7.41–7.45)		7,43 (7.4–7.45)	*p =* 0,0935 (time) *p =* 0.3506 (SBT outcome) *p =* 0.6891 (interaction)
EtCO2, mmHg	38 (36–41)	38 (37–43)	39 (35–41)	*p =* 0.0003 (time) *p =* 0.0796 (SBT outcome) *p =* 0.0600 (interaction)

*P/F, pO_2_/FiO_2_ ratio; EtCO_2_, End tidal CO_2_*.

### Electrical Activity of Respiratory Muscles During SBT

Considering muscles groups in a separate fashion, both the diaphragm EA (EAdi_surf_) and sum of accessory muscles (ACC_surf_) increased during SBT (*p* < 0.001 for the time effect).

**Figure 2:**
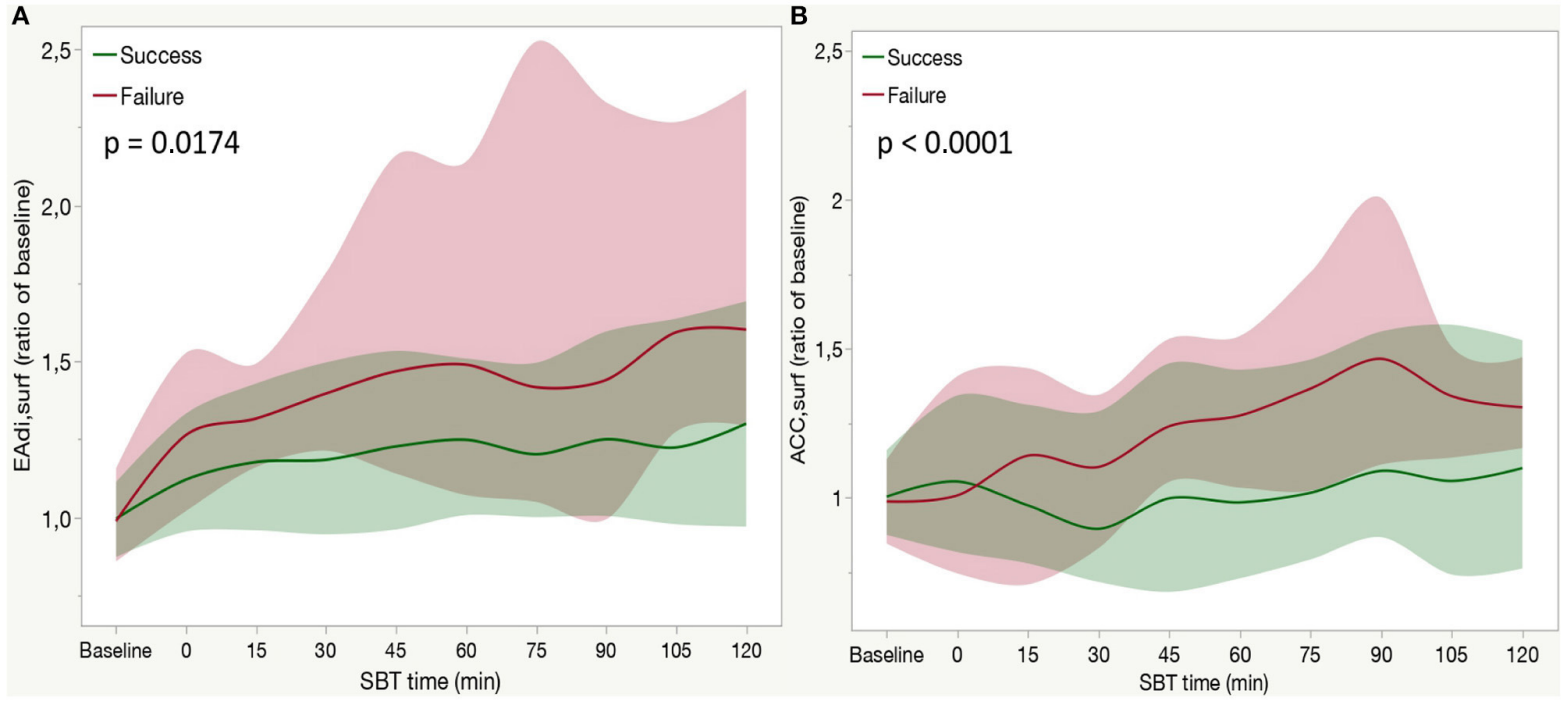
Time course of the diaphragm electrical activity [EADI_surf_ **(A)**] accessory muscle electrical activity [ACC_surf_, **(B)**] during a spontaneous breathing trial (SBT) in weaning failure (red line) or success (green line) patients. Each line indicates the median value at each time point with bands indicating interquartile range (IQR). A *P*-value refers to the between-group comparison performed with the use of the two-factor analysis of variance to test time (from baseline to 120 min of SBT) and group effects (SBT failure or success). In a *post-hoc* analysis median EAdi_surf_ at each time point was higher than baseline (*p* < 0.05) both in the failure and success group. In a *post-hoc* analysis median ACC_surf_ was higher than baseline in the success group at 75 (*p* = 0.0093), 90 (*p* < 0.0001), 105 (*p* < 0.0001), and 120 min (*p* < 0.0001) and at 75 (*p* = 0.0003), 90 (*p* < 0.0001), and 105 min (*p* < 0.0035) in the failure group.

The increase of EAdi_surf_ was higher in failure patients compared with success (*p* = 0.0174 for the interaction of time and SBT outcome, Figure 2A). ACC_surf_ increase was higher in failure patients (*p* < 0.001 for the interaction of time and SBT outcome, Figure 2B).

In particular, EAdi_surf_ started to increase at the beginning of the trial and then reached a stable plateau in the second half of the SBT. Such a pattern was present in both the groups, with a higher increase in failure patients (see *post-hoc* analysis in Figure 2A). Conversely, the increase in ACC_surf_ occurred in the second phase of SBT (after 75 min of CPAP) both in failure and success groups (see *post-hoc* analysis in Figure 2B).

### Expiratory Muscles Activity During SBT

**Figure 3:**
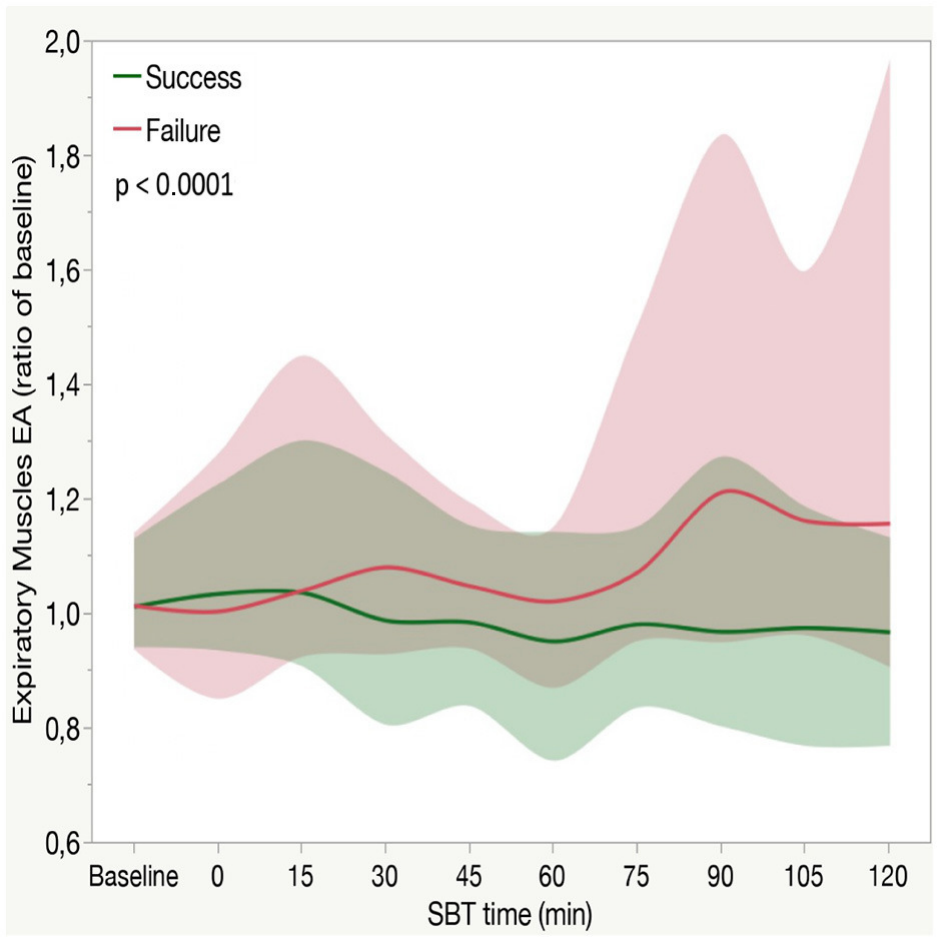
Expiratory muscles electrical activity (EXP_surf_) in patients who fail or succeed weaning attempt along with aSBT. Each line indicates the median value with bands indicating IQR. The *P*-value expressed in the figure is for the between-group comparison performed with the use of the two-factor analysis of variance to test time (from baseline to 120 min of SBT) and group effects (SBT failure or success). In a *posthoc* analysis median EXP_surf_ at each time point (from 0 to 120 min) was not different than baseline (*p* > 0.05) in the success group, while it was higher than baseline in the failure group from 75 to 120 min (*p* < 0.0001). At each time point, EXP_surf_ was higher in the failure group compared with the success group from 75 to 120 min (*p* < 0.0001).

Electrical activity of expiratory muscles (EXP_surf_) increased during SBT (*p* < 0.0001 for time effect), more in failure patients than in weaned ones (*p* < 0.0001 for the interaction between time and SBT outcome, Figure 3). Expiratory muscles activity remained stable through the entire duration of the trial in successfully weaned patients, while increased after 75 min in the failure group (see *post-hoc* in Figure 3).

### Accessory Muscles Recruitment During SBT

The ratio between accessory muscles EA relative increase and diaphragm EA relative increase (A/D ratio) represents the rate of increase in accessory muscles activity for each unit of increase in the diaphragm activity. Ultimately, it represents the accessory muscles' relative recruitment rate. A/D_ratio_ during the entire SBT was, in the whole study population, 0.83 (0.56–1.10), with no differences between failure or success group (*p* = 0.0697) (Table 2). A/D_ratio_ decreased during SBT in the success group, while it remained stable in the failure group until 90 min when it became higher than baseline (*p* < 0.0001 for effect of time, *p* < 0.0001 for interaction between time and SBT outcome). The maximum value of A/D_ratio_ of 5.39 was reached after 90 min of SBT in a failure patient (See Supplementary Material Table 2).

### Neuroventilatory Efficiency Calculated by Means of sEMG

**Figure 4:**
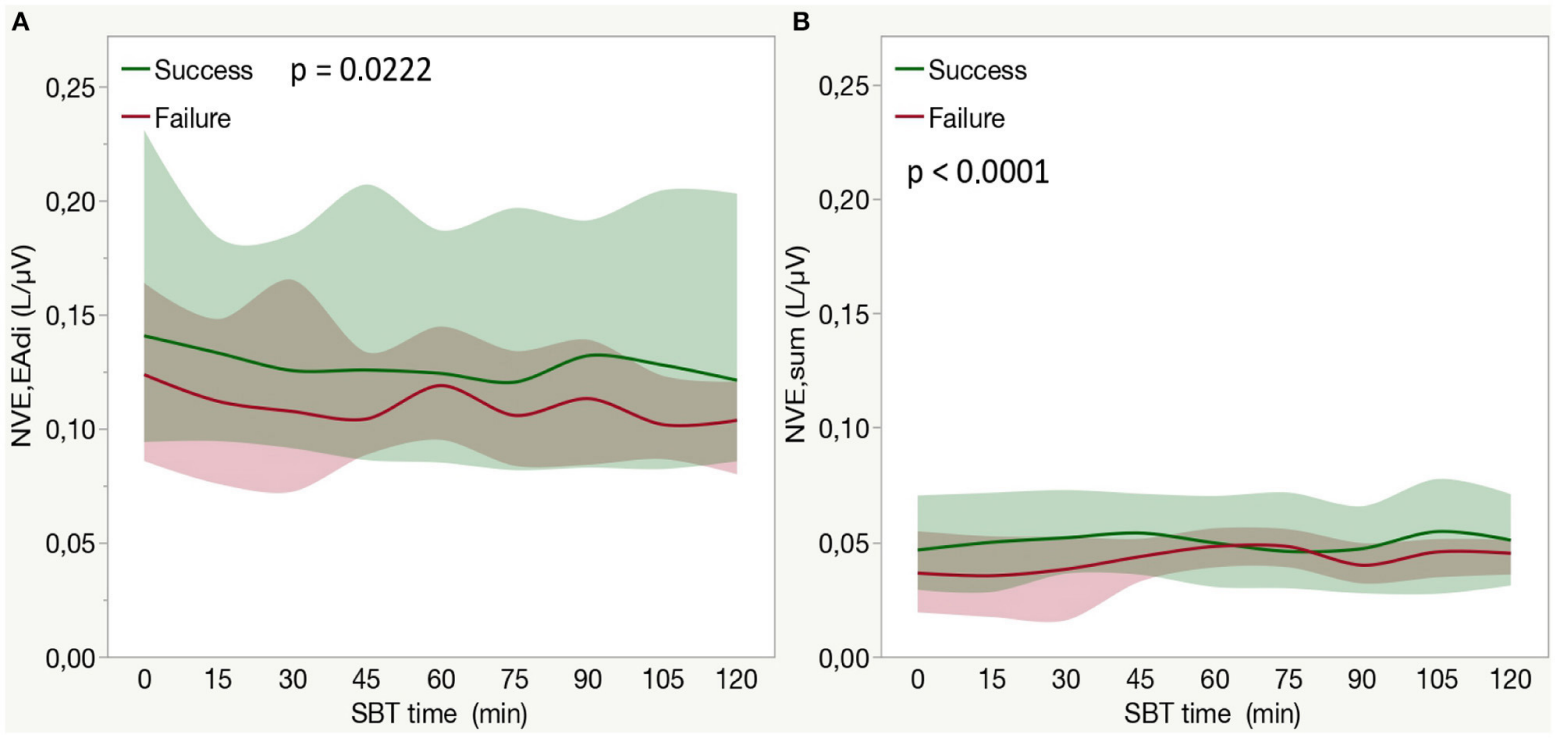
**(A)** Neuroventilatory efficiency for diaphragm surface electrical activity (NVE_EAdi_) in patients who fail or success weaning attempts among the entire duration of a SBT. Each line indicates the median value with bands indicating IQR. The *P*-value is for the between-group comparison performed with the use of the two-factor analysis of variance to test time (from 0 to 120 min of SBT with the exclusion of baseline step) and group effects (SBT failure or success). In a *post-hoc* analysis median NVE_EAdi_ at each time point (from 0 to 120 min) was not different from baseline (*p* > 0.05) both in the success and failure group. **(B)** Neuroventilatory efficiency for sum of inspiratory muscles surface electrical activity (NVE_sum_) in patients who fail or succeed weaning attempt during the entire duration of a SBT. Each bar indicates the median value with bands indicating IQR. The *P*-value is for the between-group comparison performed with the use of the two-factor analysis of variance to test time (from 0 to 120 min of SBT with the exclusion of baseline step) and group effects (SBT failure or success). In a *post-hoc* analysis comparing median NVE_sum_ at each time point with the beginning of SBT (0 min) NVE_sum_ was higher at 30 min (*p* < 0.0001), 45 min (*p* < 0.0001), and 105 min (*p* = 0.0006) in the success group, while it was higher at 60 min (*p* = 0.005) but lower at 90 min (*p* < 0.0001). NVE_EAdi_, neuroventilatory efficiency for diaphragm surface electrical activity; NVE_sum_, neuroventilatory efficiency for sum of inspiratory muscles surface electrical activity; SBT, spontaneous breathing trial.

In the whole population, considering the entire duration of the CPAP trial (from 0 to 120 min) NVE_sum_, but not NVE_EAdi_, increased during SBT (*p* < 0.0001 for effect on time). An interaction between SBT time and SBT outcome was observed for both NVE_sum_ (*p* < 0.0001, Figure 4B) and NVE_EAdi_ (*p* = 0.0222, Figure 4A). A *post-hoc* analysis revealed as NVE_EAdi_ remained stable over time, while NVE_sum_ showed a significant reduction in the second phase of SBT in failure patients (see Figure 4 for *post-hoc* analysis).

## Discussion

In this prospective crossover physiological study, we explored the role of sEMG in assessing the contribution of inspiratory and expiratory muscles during a SBT. Furthermore, we investigated differences in sEMG signal in patients who succeeded or failed the weaning attempt.

The main findings of this study can be summarized as follows:

1. Non-invasive respiratory muscles monitoring by sEMG was feasible in almost all enrolled patients, and allowed to detect surface EA of the diaphragm, accessory, and expiratory muscles.
2. Respiratory muscles EA increased during SBT, regardless of SBT outcome, and patients who failed the SBT had a higher increase of all inspiratory muscles EA compared with the patients who passed the SBT.
3. Accessory muscles recruitment was associated with SBT failure.
4. Expiratory muscles activation was detected, and it increased in patients who failed the SBT.

Respiratory muscles dysfunction is considered as one of the main causes of difficult weaning and the role of extradiaphragmatic muscles has been recently highlighted (5). We previously described the feasibility of sEMG in monitoring EAdi (15). Compared to the transdiaphragmatic measurement based on a nasogastric tube, sEMG is not only far less invasive but also widens the possibility to study extradiaphragmatic muscles.

In this work, we studied the hypothesis that accessory and expiratory muscles activation occurs when the diaphragm alone cannot further cope with a disproportionate increase in inspiratory load. Moreover, we used a SBT as a reliable model of work of breathing increase in the mechanically ventilated patients (18).

### Diaphragm EA Increase During SBT

Diaphragm EA is considered the best indicator of the neural respiratory drive (19). In our study the diaphragm EA increased rapidly when SBT started and reached a higher plateau in failure patients. This is consistent with the described increase in respiratory effort during SBT and its association with weaning failure (18, 20).

In this study, the diaphragm EA started to increase immediately after patients were switched from PS to CPAP and reached a plateau in the early phase of the trial. Liu et al. (10) studied weaning patients with a CPAP trial lasting 30 min and found similar results. Such a pattern (i.e., a steep increase that rapidly plateaus) was not observed with different weaning protocols (18) in which the increase in the respiratory drive could be lower and less steep.

According to the previous findings, we showed that the respiratory drive increases mainly in weaning failure patients during an SBT and occurs briefly after the respiratory load rises.

### Accessory Muscles Activation During SBT

Accessory muscles recruitment is a well-known clinical sign of impending muscular exhaustion and has been described during loaded breathing in the healthy subjects and intubated patients using different technologies (6–8, 13). More recently, Roesthuis et al. studied accessory muscles sEMG activity in intubated patients during a stepwise reduction of ventilatory assistance (21). The authors reported a weak correlation between the diaphragm and extradiaphragmatic EA, regardless of the level of assistance. This suggests that the increase of inspiratory load may not have a uniform effect on the different respiratory muscles, as already demonstrated in the healthy subjects (4).

In this study, weaning failure patients were characterized by a progressive increase in accessory muscles EA that reached a later peak than the diaphragm. After removing the ventilator assistance, the neural centers increase their output to the diaphragm to strengthen its contraction. During this phase, rapid shallow breathing intervenes to compensate for the disproportionate inspiratory load that the diaphragm cannot manage and accessory muscles are activated to contribute to the inspiratory work. In our patients, the EA of accessory muscles was evident in patients who failed the SBT and kept increasing throughout the trial. Accessory muscles recruitment, expressed as the amount of accessory muscle activation for each unit of diaphragm activation, shows a differential behavior in patients who failed or passed the trial. In both the groups, A/D_ratio_ decreased at the beginning of the trial, parallel to the prevalent increase in diaphragm EA. Then, in the failure patients, accessory muscles activity started to grow more than Diaphragm activity (up to 5-fold).

This pattern of respiratory muscles recruitment after an increase in respiratory load (i.e., the immediate activation of the diaphragm followed by the chest wall muscles and subsequently by expiratory muscles) is consistent with the hierarchical activation pattern previously proposed (6). In this study, we observed that accessory muscles recruitment is associated with weaning failure which is associated with a significant reduction of the force-generating capacity of respiratory muscles, as reported previously (2).

### Neuromechanical Properties of Whole Inspiratory Muscles

A limitation of diaphragm EA (EAdi) measurement is that it does not provide any information on the effective pressure generated by the diaphragm. Dres et al. (22) studied the neuroventilatry efficiency (NVE)—which is the ratio between TV and EAdi—in the patients undergoing SBT. The authors reported a severe impairment of NVE in the patients who failed weaning. Similar findings were reported by Liu et al. (10). In our patients, we observed a progressive overall reduction of diaphragm NVE that was more pronounced in failure patients.

Altogether these results suggest two important clinical points:

First, they confirm the described association between the reduction of diaphragm force-generating capacity in the presence of a disproportionate increase of respiratory load (5, 10, 22) and weaning failure. This reduction in the diaphragm neuromechanical properties may be because of the diaphragm weakness and impaired respiratory mechanics (23). However, other factors such as dynamic hyperinflation and intrinsic PEEP (24) development may act similarly. Although we could not rule out the contribution of intrinsic PEEP to weaning failure, Doourdin et al. reported that it did not differ between patients who failed and succeeded in a weaning attempt (5).

**Figure 5:**
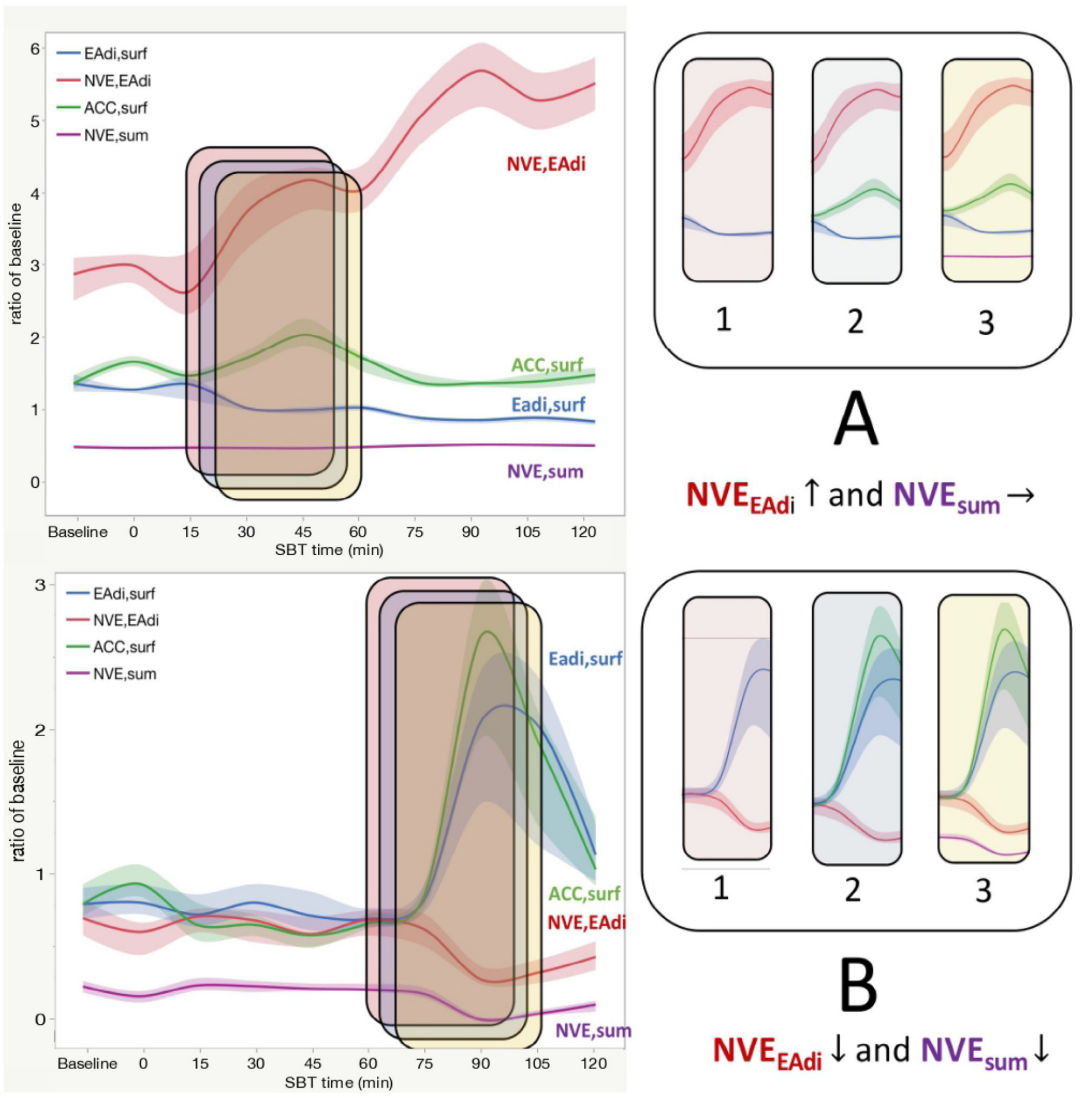
For two representative patients (**A,B** on the left) the diaphragm Electrical Activity (EAdi_surf_), accessory muscles Electrical Activity (ACC_surf_), Neuroventilatory Efficiency of the diaphragm (NVE_EAdi_), and of the whole inspiratory muscles (NVE_sum_) is shown during SBT. Lines indicated median values with bands indicating IQR. On the right side, a little time frame is sampled and Eadi_surf_, NVE_Eadi_, ACC_surf_, and NVE_sum_ are added stepwise (from frames 1 to 3) to illustrate the different behavior of this variable, in particular of NVE_EAdi_ and NVE_sum_. **(A)**. In this representative patient who fails SBT, a progressive increase in NVE_EAdi_ (red line) together with a stable EAdi_surf_ (blue line) is observed at the beginning of the trial (Panel 1). This can be interpreted with a prognostically good improvement of the diaphragm mechanical properties since it can produce more mL of tidal volume (TV) for each microV of EA. But if we look at panel 2, we see as accessory muscles EA increases (green line). The accessory muscles hiddenly ≪help≫ the diaphragm and this results in a larger TV. If we compute NVE taking into account all inspiratory muscles, it remains stable (panel 3). This is a case in which an apparent improvement in the neuromechanical properties of the diaphragm is falsely observed in presence of accessory muscles recruitment when it is calculated with the only diaphragm EA. **(B)** In this representative patient who success SBT, a steep increase in diaphragm EA (EAdi_surf_, blue line) is observed at the end of the CPAP trial (panel 1), together with a reduction in NVE_EAdi_ (red line). This circumstance can be interpreted as a worsening in the diaphragmatic performance at the end of SBT, eventually because of the muscle fatigue and exhaustion, leading to a reduction in force (and tidal) generating capacity of the diaphragm. If we take into account accessory muscles, we can see as their EA increases parallel to the diaphragm (green line, panel 2). So, if NVE is calculated taking into account all inspiratory muscles (NVE_sum_, purple line) it decreases in the same way as that calculated only for the diaphragm (panel 3). In this case, EA and mechanical properties of the diaphragm and accessory muscles vary in the same manner, and diaphragm monitoring can be representative of the whole respiratory muscles. EAdi_surf_, diaphragm surface electrical activity; ACC_surf_, accessory muscles surface electrical activity; NVE_EAdi_, neuroventilatory efficiency for EAdi_surf_; NVE_sum_, neuroventilatory efficiency for ACC_surf_.

Second, NVE_EAdi_ considers the only contribution of the diaphragm EA, and it could overestimate its force-generating capacity if accessory muscles contribute to the generation of TV. This situation is conceptualized by means of two representative patients in Figure 5.

For these reasons, the lack of assessment of the accessory inspiratory muscles may limit a careful interpretation of the neuromechanical coupling in patients undergoing SBT.

### Expiratory Muscles Activity During SBT

In the presence of an imbalance between inspiratory load and inspiratory muscles capacity, expiratory muscles recruitment occurs. Activation of the abdominal wall muscles has different physiological consequences on the respiratory dynamics. First, as the diaphragm is relaxed during the expiratory phase, the activation of expiratory muscles reduces the transpulmonary pressure and promotes active deflation of the lungs by increasing abdominal and pleural pressure. This might decrease the end-expiration volume below functional residual capacity. In turn, the elastic recoil stored in the chest wall will act as an adjunctive inspiratory force to start the next inspiration. Second, the increased intra-abdominal pressure moves the relaxed diaphragm to a more cranial position that is mechanically more advantageous in terms of muscle length–tension relation.

However, expiratory muscles recruitment may also exert detrimental effects in mechanically ventilated patients, as the expiratory reduction of transpulmonary pressure may lead to atelectasis and airway closures (23).

Expiratory muscles activation has been observed during weaning from mechanical ventilation (6, 25) in failure patients. Doorduin et al. demonstrated that the pressure generated by expiratory muscles increased during a weaning trial and it was strongly associated with weaning failure (5).

In our investigation, we observed a similar pattern of progressive expiratory muscles recruitment in the late phase of the SBT, with a strong interaction between time and trial outcomes. In addition, we demonstrate an unequivocal electromyographic activity of the rectus abdominis that reflects an expiratory muscle activity.

However, we specifically measured EA and not the force effectively generated by the expiratory muscles. Since many studies have demonstrated that the respiratory muscles weakness involves accessory and expiratory muscles (23, 26, 27), this issue deserves further investigation.

The strong association between weaning failure and the amplitude of such extradiaphragmatic muscle activity may indicate that this phenomenon is driven by the respiratory muscle exhaustion in relation to a disproportionate increase in work of breathing during weaning.

### Strength and Limitations

This is one of the first report that provide a detailed description of the diaphragm and extradiaphragmatic recruitment patterns in the weaning patients using sEMG. Although the sample size is somehow limited, the number of respiratory cycles analyzed is extremely high (up to 7,000, about 200 cycles for each patient) and it represents one of the main strengths of our work. Indeed, the prolonged duration of SBT, compared with that of the similar studies, may have allowed to explore a different kinetic of muscle recruitment following a progressive respiratory muscles exhaustion.

This study has some limitations. First, the measurement of the invasive diaphragm EA by the dedicated nasogastric tube and of the inspiratory effort by the use of the esophageal pressure (Pes) catheter was not available. However, we previously demonstrated a tight correlation between the sEMG signal and both invasive EAdi and Pes signal (15). Second, accessory muscles are also involved in functions other than inspiratory action, such as airway patency, head rotation, neck flexion, and trunk stabilization. This rises some concerns about the possibility to obtain a reliable sEMG signal with an adequate signal-to-noise ratio. In order to minimize this caveat, we chose two representative accessory muscles in which the role of the postural activity is likely very limited in the presence of sedation, intubation, and supine positioning in an ICU bed. Furthermore, chronic obstructive pulmonary disease patients tend to be more represented in the failure group. We cannot exclude that previous respiratory disease, and in particular, those characterized by obstructive physiology may have contributed to SBT failure and to the activation pattern of extradiaphragmatic muscle (in particular expiratory muscles) we observed. In sum, according to the exploratory nature of this study, the sample size is limited. For this reason, the investigation of the role of sEMG during SBT in a larger patient population would help to seek confirmation of the preliminary findings.

## Data Availability Statement

The raw data supporting the conclusions of this article will be made available by the authors, without undue reservation.

## Ethics Statement

The studies involving human participants were reviewed and approved by San Gerardo Hospital Ethical Committee ASST Monza. The patients/participants provided their written informed consent to participate in this study.

## Author Contributions

MP, AB, GG, GF, and GB contributed to conception and design of the study. MP, AB, and FR collected data. MP performed the statistical analysis. MP and ER wrote the first draft of the manuscript. All authors contributed to manuscript revision, read, and approved the submitted version.

## Conflict of Interest

GB was employed by Draeger Medical Italy, Draeger Medical Germany, and Pfizer. The remaining authors declare that the research was conducted in the absence of any commercial or financial relationships that could be construed as a potential conflict of interest.

## Publisher's Note

All claims expressed in this article are solely those of the authors and do not necessarily represent those of their affiliated organizations, or those of the publisher, the editors and the reviewers. Any product that may be evaluated in this article, or claim that may be made by its manufacturer, is not guaranteed or endorsed by the publisher.
