# Supplementary material for: Accessory and Expiratory Muscles Activation During Spontaneous Breathing Trial: A Physiological Study by Surface Electromyography
Source: Front Med (Lausanne). 2022 Mar 10;9:814219. doi: 10.3389/fmed.2022.814219 (PMC8965594; doi:10.3389/fmed.2022.814219)
Supplement: Supplementary file 1 [file Data_Sheet_1.docx]

**Accessory and Expiratory muscles activation during spontaneous breathing trial:**

**a physiological study by surface electromyography.**

Matteo Pozzi^1,2^, Emanuele Rezoagli^1^, Alfio Bronco^1,2^, Francesca Rabboni^1^, Giacomo Grasselli^3,4^, Giuseppe Foti^1,2^, Giacomo Bellani*^1,2^

**Affiliations**

^1^Department of Medicine and Surgery, University of Milano-Bicocca, Monza, Italy; ^2^Department of Emergency and Intensive Care, San Gerardo Hospital, Monza, Italy; ^3^Department of Anesthesia, Intensive Care and Emergency, Fondazione IRCCS Ca' Granda Ospedale Maggiore Policlinico, Milan; ^4^Department of Pathophysiology and Transplantation, University of Milan, Milan, Italy

**Supplementary material**

**Off-line data analysis .**

Respiratory Rate (RR) and Rapid Shallow Breathing Index (RBSI) were calculated according to standard practice.

Respiratory System Statist Compliance (Cpl,rs), was calculated during an inspiratory hold at baseline, after 1 hour of SBT and at the end of SBT (after 2 hours or, in failure patients, whenever it occurred). At the same points p0.1 was calculated during a brief expiratory occlusion by means the dedicated tool on the ventilator, and EtCO_2_ was measured by means sidestream volumetric capnography.

Blood Gas Analysis (BGA) for pO_2_, pCO_2_ and pH was collected at baseline and at the end of SBT (after 2 hours or, in failure patients, whenever it occurred). FiO_2_ was collected to calculate the ratio between pO_2_ and FiO_2_ (P/F).

**Spontaneous Breathing Trial (SBT) duration and sampled respiratory cycles in success and failure patients.**

Patients underwent a SBT lasting two hours, if interruption criteria were not first met. In case of successfully termination of SBT, a total amount of 220 respiratory cycles were manually sampled (see Methods): Forty respiratory cycles during baseline step and nine clusters of twenty respiratory cycles (at 0; 15 ;30 ;45 ;60 ;75 ;90 ; 105 and 120 minutes) during entire SBT.

If SBT was prematurely terminated, the amount of sampled respiratory cycles was proportional to SBT duration, being they sampled at the same time points. Table 1 resume the amount of sampled respiratory cycles for success and failure patients.

**Table 1**. Sampled respiratory Cycles in failure and success patients

|  | **Time points** | **Total respiratory cycles** |
| --- | --- | --- |
| **Success Group (n = 29)** |  |  |
| All patients | Baseline + 9 | 220 cycles |
|  |  | **TOT: 6380 cycles** |
| **Failure group (n = 8)** | | |
| PT #1 | Baseline + 3 | 99 cycles |
| PT #2 | Baseline + 7 | 178 cycles |
| PT #3 | Baseline + 3 | 100 cycles |
| PT #4 | Baseline + 4 | 120 cycles |
| PT #5 | Baseline + 3 | 100 cycles |
| PT #6 | Baseline + 2 | 80 cycles |
| PT #7 | Baseline + 6 | 160 cycles |
| PT #8 | Baseline + 2 | 80 cycles |
|  |  | **TOT: 917 cycles** |
| **TOT: 7297** | | |

The difference between the theorical and actual number of sampled cycles in patient #1 and #2 relies on few respiratory cycles excluded for quality issues.

**Table 2**. The ratio between Accessory muscles and Diaphragm Electrical Activity (A/D_ratio_) at different time points for the whole study population.

Legend: A/D_ratio_: Ratio between Accessory muscles Electrical Activity and Diaphragm Electrical Activity. IQR: Interquartile Range.
